# A rare seizure: A case of seizure-induced central acetabular fracture-dislocation in a patient with chronic kidney disease and hepatitis-B

**DOI:** 10.1016/j.tcr.2026.101322

**Published:** 2026-02-24

**Authors:** Sandeep Patel, Prasoon Kumar, Ankit Dadra, Utkarsh Reddy, Arjit Bansal

**Affiliations:** a Post Graduate Institute of Medical Education and Research (PGIMER), Chandigarh, 160012, India

**Keywords:** Seizure, Acetabular fracture, Chronic kidney disease, Hepatitis-B, Modified Stoppa approach

## Abstract

Seizure induced fractures are uncommon and usually involve long bone fractures like proximal humerus, proximal femur, shoulder dislocations etc. These occur due to intense muscle contractions or may result from indirect trauma post-seizure episode in the patient. Acetabulum fracture with central head dislocation, as seen in this case, is very rare following a seizure episode. It was further complicated by the presence of Hepatitis-B and dialysis dependent CKD both of which impact the surgical outcomes as well as pose challenges in the management of patient in the perioperative period.

**Case:**

A 32-year-old male with a history of CKD and Hepatitis-B presented with severe hip pain following a generalised tonic-clonic seizure. Imaging revealed an anterior column fracture with central dislocation of the femoral head. The patient underwent open reduction and internal fixation (ORIF) via the Modified Stoppa's approach. Perioperative management focused on optimising renal and hepatic dysfunction while minimising infection risk. Postoperatively, the patient demonstrated stable fixation and uneventful wound healing and good functional outcome.

**Conclusion:**

This case highlights the rare occurrence of seizure induced acetabulum fracture and the challenges of perioperative management of such injuries in patients with metabolic comorbidities and thus helps with understanding the interplay between seizure induced trauma, CKD complicating the bone quality thus affecting the fixation and perioperative risks in Hepatitis-B.

## Highlights

• Rare case of seizure-induced central acetabular fracture-dislocation
• Highlights patients with chronic kidney disease and hepatitis B
• Managed using the modified Stoppa approach and quadrilateral plate fixation
• Demonstrates risk of skeletal injury without external trauma
• Emphasizes early diagnosis and multidisciplinary care

## Introduction

Acetabular fractures are complex injuries typically resulting from high-energy trauma such as road traffic accidents or falls from height and associated central dislocation of femoral head are rare and usually occur when there is direct axial force transmitted through greater trochanter in abducted limb into the femoral head and through the medial wall of the acetabulum [1]. The intrapelvic displacement of femoral head may cause comminuted fractures of the anterior column and quadrilateral plate [1]. While seizure-related musculoskeletal injuries are not uncommon, often involving shoulder dislocations or vertebral compression fractures [2], fractures of the pelvis and acetabulum following seizures are exceedingly rare [3] [Table I]. These injuries may occur due to intense and uncoordinated muscular contractions during tonic-clonic episodes, even in the absence of direct trauma. Patients with chronic kidney disease (CKD) often exhibit compromised bone quality due to renal osteodystrophy, making them more susceptible to fractures and problems with bone healing in post-operative period [4]. Additionally, associated metabolic disturbances and comorbidities such as Hepatitis-B infection can complicate management due to deranged coagulation profile, thus increasing the risk of bleeding and deranged immunity, thus increasing the risk of infections [4], [5]. In this case report, we present an unusual instance of an acetabular fracture with central dislocation of femoral head following a seizure in a patient with underlying CKD and Hepatitis-B positivity. The case highlights the diagnostic challenges and surgical decision-making in managing such a rare injury pattern in a medically complex patient, managed in an apex trauma center of north India.

**Table I:** Similar case reports in literature and comparison with our study.

Author/Year	Age/Sex	Etiology of seizure	Fracture	Risk factors	Management	Outcome
1993/Berman et al. [10]	54/F	Generalised tonic clonic seizure while receiving dialysis	Unilateral central acetabular fracture-dislocation	CKD on dialysis	Conservative	Shortening + Limp – Walking without aid [time period not mentioned]
1998/Papanikolaou et al. [11]	60/F	Generalised tonic clonic seizure	Bilateral acetabular fracture with central femoral head dislocation	CKD on dialysis	Conservative	Died at 23rd day
2014/Meena et al. [12]	27/F	Neurocysticercosis Induced seizure	Bilateral central acetabular fracture dislocation	–	Conservative	Good outcome at one yr follow up
2019/Nusrat et al. [13]	13/M	Generalised tonic clonic seizure (K/c/o seizure disorder)	Bilateral acetabular fracture	AKI	ORIF	–
Our study	32/M	Generalised tonic clonic seizure	Unilateral central acetabular fracture-dislocation	CKD Hepatitis-B	ORIF	Good outcome at three months follow up

## Case study

**Fig. 1:**
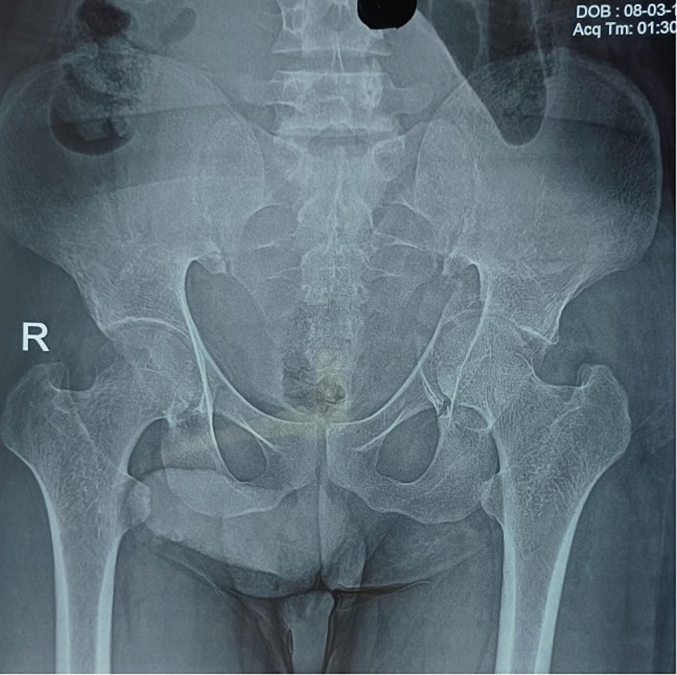
Preoperative antero-posterior view of pelvis showing left side acetabulum fracture [disruption of ilio-pectineal line indicating anterior column disruption], notice medial displacement of femoral head through the disrupted quadrilateral plate.

**Fig. 2:**
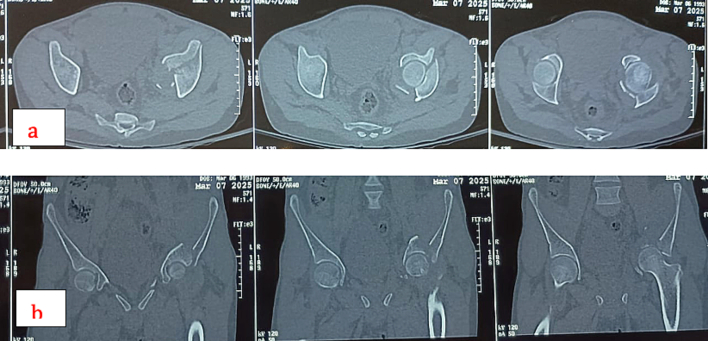
(a) Preoperative CT scan [axial sections]. (b) Preoperative CT scan [coronal sections].

**Fig. 3:**
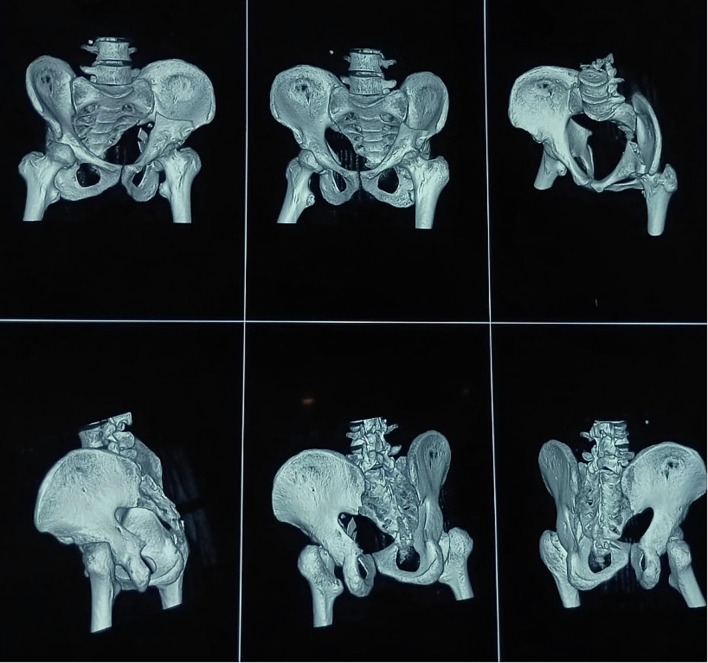
Preoperative CT scan [3D reconstruction].

A thirty-two-year-old male presented to the emergency department of our institution with a complaint of pain in the left groin region and inability to move his left lower limb after experiencing a generalised tonic-clonic seizure at home. The patient had no prior history of seizures and no evidence of external trauma or fall during the episode. The episode lasted for approximately thirty seconds, and in the immediate post-ictal phase, he did not report any confusion or loss of consciousness. He also had a history of chronic kidney disease on maintenance hemodialysis thrice a week and was also a known case of Hepatitis-B on medical treatment for four months. On examination, the left lower limb was shortened, abducted and externally rotated. There was no distal neurovascular deficit. Localised tenderness and restricted range of motion at the hip joint were noted. Plain radiographs of the pelvis revealed a fracture of the left acetabulum involving the anterior column with medial displacement of the femoral head into the pelvis (Fig. 1). A CT scan confirmed the diagnosis and delineated the fracture morphology (Fig. 2, Fig. 3). Laboratory investigations showed HBsAg: Reactive (Index Value 57.08), Serum Creatinine: 6.8 mg/dL, Urea: 78 mg/dL, Hemoglobin: 9.8 g/dL, protein/albumin: 5.3/2.9, PT-INR:1.1. Echocardiogram and chest radiograph were normal and NCCT head followed by EEG and MRI were done for evaluation of seizures. Pre-anaesthetic clearance was obtained in consultation with the nephrology, hepatology and neurology teams.

### Treatment

The patient was taken up for surgery after preoperative stabilization, which included hemodialysis and perioperative antiepileptic cover after considering drugs safety in CKD and making necessary dosage adjustments. Under general anaesthesia, open reduction and internal fixation was performed in the supine position via a Modified Stoppa's approach. Intraoperatively, the fracture was found to involve the anterior column extending into the quadrilateral plate with central displacement of the femoral head. An anatomical reduction was achieved, and a 10-hole 3.5 mm suprapectineal quadrilateral surface plate was contoured and fixed. Three screws were placed proximal to the fracture site into the ilium, and two screws were inserted into the superior pubic ramus for stabilization. Care was taken to avoid injury to the obturator neurovascular bundle and the corona mortis. The surgical procedure was uneventful. The patient was monitored in the high-dependency unit postoperatively.

### Postoperative course

**Fig. 4:**
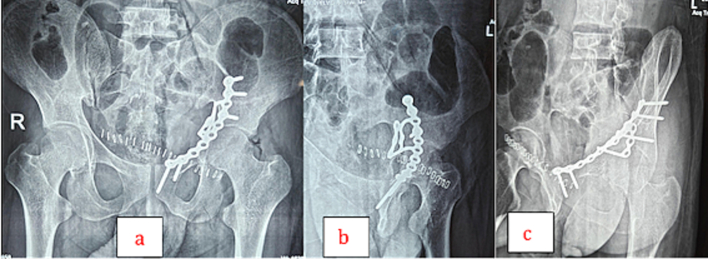
(a) Postoperative X-ray [antero-posterior view]. (b) Postoperative X-ray [iliac oblique view]. (c) Postoperative X-ray [obturator oblique view].

Postoperative radiographs confirmed anatomical reduction and stable fixation (Figs. 4). The patient was kept strictly non-weight-bearing on the operated limb for six weeks, followed by gradual weight-bearing as tolerated under physiotherapy supervision. Antiviral precautions and strict aseptic techniques were maintained throughout hospitalisation due to Hepatitis-B. He underwent routine hemodialysis during the postoperative period. Wound healing was uneventful, and there were no signs of infection or complications. After wound inspection and antiseptic dressing of the suture line, he was discharged from the hospital.

**Fig. 5:**
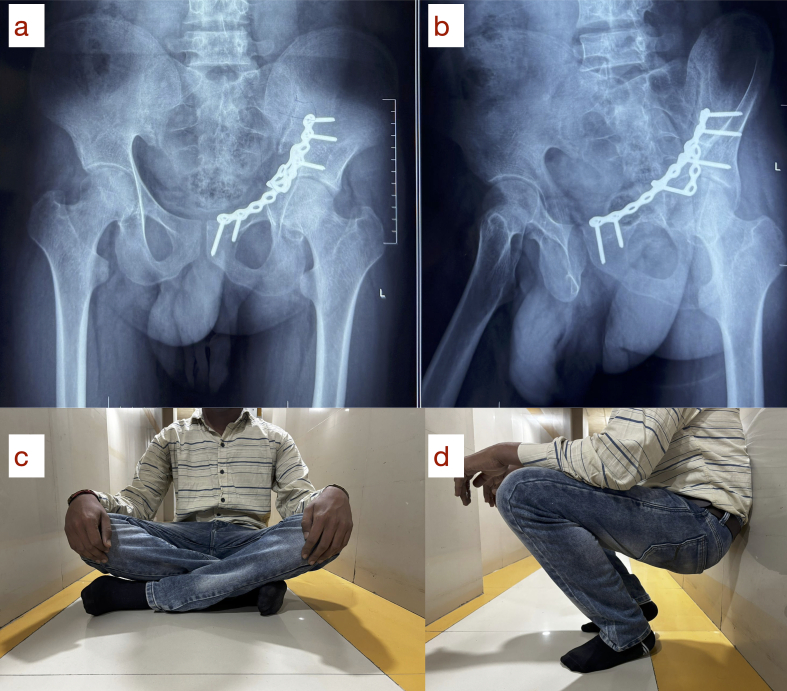
One-year follow-up radiographs demonstrating complete fracture union: (a) anteroposterior view and (b) obturator oblique view. Corresponding clinical photographs showing full functional recovery with the ability to (c) sit cross-legged and (d) squat.

At the 1-year follow-up, the patient was pain-free, ambulatory without support, and showed radiographic signs of fracture union with no hardware complications (Fig. 5-a, b).

## Results

At 1-year of follow up patient showed clinical and radiological union (Fig. 5-a, b), was able to walk without support. The Harris Hip Score was 96, and the range of motion on the left was comparable to the normal side. The patient was able to do all his routine activities, sit with crossed legs and squat (Fig. 5-c, d).

## Discussion

Acetabular fractures are rare and often missed in the immediate post-seizure phase due to altered consciousness or nonspecific complaints. In patients with compromised bone quality, such as those with renal osteodystrophy, even minimal trauma or muscle contractions can lead to significant fractures. The massive forces generated by contraction of the hip musculature, particularly the iliopsoas, gluteal muscles, and adductors, may have led to this type of fracture pattern in this patient, resulting in a central acetabular fracture-dislocation. There are a few case reports with this mechanism in the literature [Table I].

### Surgical considerations

The modified Stoppa approach allows excellent access to the anterior column and quadrilateral plate. It offers minimal soft tissue disruption and a direct view of the medial wall [9]. In this case, the use of a 10-hole 3.5 mm quadrilateral surface plate provided the necessary stability, and its fixation across both the ilium and pubis ensured robust support.

### Role of comorbidities

CKD is associated with a spectrum of bone disorders collectively known as chronic kidney disease–mineral and bone disorder (CKD-MBD) [4], which includes osteomalacia, secondary hyperparathyroidism, and adynamic bone disease. These conditions result in decreased bone mineral density and structural integrity, predisposing patients to low-impact or even non-traumatic fractures, especially in the pelvis and spine and delayed bone healing [4]. In this patient, the seizure-induced muscular forces alone were sufficient to cause a complex acetabular fracture-dislocation, which would be highly unusual in a healthy individual. Hepatitis-B positivity, while not directly implicated in the mechanism of injury, complicates perioperative management. It necessitates careful infection control practices, especially during surgical intervention [7], [8]. Additionally, hepatic involvement in chronic Hepatitis-B can impair clotting function and affect the metabolism of anaesthetic and analgesic agents. It also limits the use of certain medications and requires vigilant monitoring of liver function in the postoperative period, thus needing longer hospital stays [7], [8].

## Conclusion

Acetabular fracture with central dislocation of the femoral head following a seizure is an extremely rare presentation. In patients with metabolic bone disease or CKD, the skeletal system may be more vulnerable to injury even without external trauma. A high index of suspicion, especially in the post-ictal period when unexplained limb pain or immobility is noted, should prompt early radiological evaluation. Early recognition and management are crucial to prevent long-term morbidity.

## CRediT authorship contribution statement

**Sandeep Patel:** Investigation, Funding acquisition, Formal analysis, Data curation, Conceptualization. **Prasoon Kumar:** Project administration, Methodology, Formal analysis, Conceptualization. **Ankit Dadra:** Writing – review & editing, Writing – original draft, Visualization, Supervision, Software, Conceptualization. **Utkarsh Reddy:** Writing – review & editing, Writing – original draft, Visualization. **Arjit Bansal:** Writing – review & editing, Writing – original draft, Resources, Investigation, Conceptualization.

## Patient consent

Written informed consent was obtained from the patient for publication of this case report and accompanying images. A copy of the written consent is available for review by the Editor-in-Chief of this journal.

## Ethical approval

Ethical clearance for this case report was obtained from the institutional ethics committee.

## Funding

This research did not receive any specific grant from funding agencies in the public, commercial, or not-for-profit sectors.

## Declaration of competing interest

The authors declare no conflict of interest.
